# Inhibiting avian influenza virus shedding using a novel RNAi antiviral vector technology: proof of concept in an avian cell model

**DOI:** 10.1186/s13568-016-0187-y

**Published:** 2016-02-24

**Authors:** Lyndsey M. Linke, Jeffrey Wilusz, Kristy L. Pabilonia, Johannes Fruehauf, Roberta Magnuson, Francisco Olea-Popelka, Joni Triantis, Gabriele Landolt, Mo Salman

**Affiliations:** Animal Population Health Institute, Colorado State University, Fort Collins, CO USA; Department of Clinical Sciences, Colorado State University, Fort Collins, CO USA; Department of Microbiology, Immunology and Pathology, Colorado State University, Fort Collins, CO USA; Cambridge Biolabs, Cambridge, MA USA; Environmental Health Services, Colorado State University, Fort Collins, CO USA

**Keywords:** Avian influenza, siRNA delivery, Transkingdom RNAi, Bacterial vector, Influenza antiviral

## Abstract

Influenza A viruses pose significant health and economic threats to humans and animals. Outbreaks of avian influenza virus (AIV) are a liability to the poultry industry and increase the risk for transmission to humans. There are limitations to using the AIV vaccine in poultry, creating barriers to controlling outbreaks and a need for alternative effective control measures. Application of RNA interference (RNAi) techniques hold potential; however, the delivery of RNAi-mediating agents is a well-known obstacle to harnessing its clinical application. We introduce a novel antiviral approach using bacterial vectors that target avian mucosal epithelial cells and deliver (small interfering RNA) siRNAs against two AIV genes, nucleoprotein (NP) and polymerase acidic protein (PA). Using a red fluorescent reporter, we first demonstrated vector delivery and intracellular expression in avian epithelial cells. Subsequently, we demonstrated significant reductions in AIV shedding when applying these anti-AIV vectors prophylactically. These antiviral vectors provided up to a 10,000-fold reduction in viral titers shed, demonstrating in vitro proof-of-concept for using these novel anti-AIV vectors to inhibit AIV shedding. Our results indicate this siRNA vector technology could represent a scalable and clinically applicable antiviral technology for avian and human influenza and a prototype for RNAi-based vectors against other viruses.

## Introduction

Avian influenza virus (AIV) outbreaks in poultry have led to the global culling of millions of birds and the net loss of billions of dollars. These outbreaks are of concern, not only because of the degree of virulence observed in poultry resulting in severe economic consequences, but also due to the potential transmission to mammalian species, including humans.

Effective prevention measures must be available to prepare for potential outbreaks, however, current vaccination strategies for birds are limited. The most efficacious vaccines must be administered subcutaneously or intramuscularly (Halvorson 2008; Rudolf et al. 2010; Swayne 2012), an impediment to successfully immunizing large numbers of poultry in a short period. In the naïve bird, protective antibody production takes two to 3 weeks to acquire following vaccination (Kim et al. 2009). Frequently, improper storage and handling leads to vaccine failure (Swayne and Kapczynski 2008). To elicit efficient protection, the vaccine must be HA-subtype specific to the outbreak virus (Arzt et al. 2010; Bennink and Palmore 2004; Escorcia et al. 2008; Swayne 2012; Zhou et al. 2008). Over time, stockpiles of vaccines become obsolete and new vaccines must be generated. These limitations convey a genuine need to develop a prophylactic that would offer universal protection against any subtype or strain of AIV and would provide rapid protection in the face of an outbreak.

Using RNAi to develop antivirals has created a wealth of research focused on controlling diseases using siRNAs (Barik and Lu 2015; Chen et al. 2009; DeVincenzo 2012; Long et al. 2010; Lyall et al. 2011; Shi et al. 2012). Others have previously demonstrated that siRNA mediated knockdown targeting both the viral NP and PA genes significantly inhibits influenza replication (Ge et al. 2004; Khantasup et al. 2014; Stoppani et al. 2015; Tompkins et al. 2004; Zhou et al. 2007, 2008). However, the challenge of intracellular delivery of these RNAi-mediating agents has historically been an obstacle to harnessing their capabilities and clinical application (Li and Shen 2009). SiRNAs require a delivery vehicle, and two examples are genetically engineered viruses and synthetic carriers (Aigner 2009; DeVincenzo 2012; Ge et al. 2004; Li et al. 2006). However, these viral delivery vectors can pose significant concerns for clinical efficacy, including associated hepatotoxicity and tumorigenesis (Beer et al. 2010; Davidson and McCray 2011; Ge et al. 2004; Grimm et al. 2006; Hacein-Bey-Abina et al. 2008). Past reports warn of off-target effects from short hairpin RNA (shRNA) viral vectors resulting in cell death and organ problems in transgenic animals (Grimm et al. 2006). Finally, synthetic siRNA carriers have low delivery efficiencies and require higher doses, which is not only cost prohibitive, but often toxic (DeVincenzo 2012; Ge et al. 2004).

The aim of this study was to investigate the inhibition of AIV shedding in avian epithelial cells using the RNAi delivery platform, transkingdom RNAi (tkRNAi). TkRNAi uses nonpathogenic *E. coli* engineered to transcribe shRNA from a plasmid (pmbv43) that also encodes two factors allowing shRNA delivery to mucosal epithelial cells; the invasin gene (*Inv*) and the listeriolysin O gene (*hylA*). The *Inv* gene is necessary for expression of invasin protein on the *E. coli* surface (Xiang et al. 2006), which interacts with β(1) integrin receptors present on mucosal epithelial cells resulting in receptor mediated endocytosis (Conte et al. 1994; Isberg and Barnes 2001; Isberg and Leong 1990). The *hlyA* gene encodes a pore-forming toxin that facilitates the rupture of the endosomal membrane and the subsequent release of shRNA into the cell’s cytoplasm (Grillot-Courvalin et al. 1998; Mathew et al. 2003; Nguyen and Fruehauf 2009; Radford et al. 2002; Xiang et al. 2006). The tkRNAi vector is a diaminopimelic acid (Dap) auxotrophic mutant and is kanamycin resistant.

**Fig. 1:**
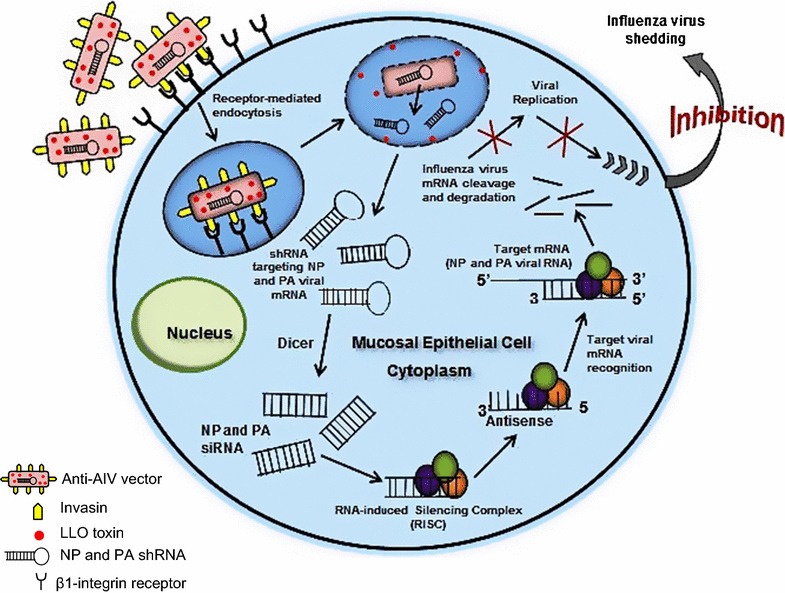
Diagrammatic representation of the anti-AIV vector’s mechanism of action against AIV in an avian epithelial cell

Figure 1 provides a diagrammatic picture of the anti-AIV vector’s mechanism of action in an avian epithelial cell and its ability to inhibit infectious AIV shedding. We designed tkRNAi vectors (anti-AIV vectors) that constitutively generate shRNAs targeting conserved sequences within the NP and PA viral genes, with the purpose to demonstrate in vitro proof-of-concept for using these anti-AIV vectors to inhibit AIV shedding in an avian cell model. These anti-AIV vectors would offer several advantages over conventional AIV vaccines and improve upon existing RNAi antiviral approaches for several reasons. These vectors are engineered to target mucosal epithelium, so are ideal for targeted delivery aimed at preventing respiratory diseases. As non-conjugative vectors, they will not integrate into the host genome, eliminating the risk for tumorigenesis. Furthermore, the tkRNAi platform has a confirmed safety record for clinical purposes (Nguyen and Fruehauf 2009; Xiang et al. 2006, 2009), and as non-conjugative, non-pathogenic and non-colonizing bacteria, do not pose any known risk to the host. Although these vectors target viral sequences that have high levels of conservation observed over time and geographic location to limit the risk for viral escape, they can be quickly adapted to respond to viral mutation. Unlike conventional vaccines, this antiviral vector would reduce trade barriers associated with reactivity of vaccinated animals because animals would not test positive for AIV antibodies. These vectors could be administered intranasally, orally, or as an aerosol suitable for inhalation. Finally, these anti-AIV vectors could be generated in a scalable process for low-cost production, stockpiled for long-term use, and applied rapidly via mass aerosolization.

## Methods

### Cells and viruses

Chicken primary hepatocellular carcinoma epithelial (LMH) cells (ATCC CRL-2117) were maintained in Waymouth’s MB 752/1 medium (Life Technologies, Carlsbad, USA), with 10 % heat inactivated fetal bovine serum (FBS), 2 mM l-glutamine, 100 Units/mL penicillin and 1 mg/mL streptomycin (Life Technologies), and unless stated otherwise, incubated at 37 °C in the presence of 7 % CO_2_. All LMH culture vessels were pre-coated with 0.1 % gelatin (EMD Millipore Corporation, Billerica, USA). Madin-Darby canine kidney (MDCK) cells (ATCC CCL-34) were used to quantify infectious viral titers for all infection assays. MDCK cells were grown in minimum essential medium with Earle’s balanced salts (MEM/EBSS), 2 mM l-glutamine, 10 % FBS, 0.5 % sodium pyruvate (100 mM solution, Life Technologies), 0.5 % MEM non-essential amino acids (NEAA) (100 × solution, Life Technologies), and 100 Units/mL penicillin and 1 mg/mL streptomycin at 37 °C in the presence of 7 % CO_2_.

A/chicken/Texas/473-2/2010(H6N2) and A/turkey/Colorado/235497/2003(H8N4) were propagated in LMH cells. H6N2 and H8N4 strains represented two AIV subtypes isolated from poultry and previously replicated to high titers in LMH cells with visual cell damage indicative of a cytopathic effect (CPE) at 48 h post infection (hpi). CPE was defined as observed destruction of the cell monolayer after fixing and staining cells with crystal violet. Viral stocks were generated in LMH cells and stored at −80 °C. Working virus stocks and all experimentally derived virus titers were measured by 50 % tissue culture infectious dose (TCID_50_) assay in MDCK cells. Optimal multiplicity of infection (MOI) was empirically derived based on the lowest MOI that produced CPE in LMH cells within 48 hpi.

### Construction of shRNA vectors

Sense and antisense RNA sequences for NP and PA were previously published (Ge et al. 2003), and were used to construct appropriate shRNAs. Using the BLAST heuristic algorithm to find the degree of sequence alignment, siRNA sense strand complementarity to the NP and PA viral gene sequences associated with H6N2 and H8N4 were verified. The NP and PA siRNA sequences both aligned against the corresponding NP and PA sequences from H8N4 and H6N2 subtypes with 100 % identity (Table 1). GenBank accession numbers for the NP and PA viral genes are given in Table 2. To decrease the possibility of off-target silencing, both 19 nt siRNA sequences were analyzed by BLAST search to ensure a lack of significant sequence complementarity to any known chicken gene sequence. A key feature of siRNA recognition involves perfect complementary base pairing between the siRNA seed region (nucleotides 2–8) on the antisense 5′ end and the mRNA target gene (Carthew and Sontheimer 2009; Ma et al. 2005; Martinez et al. 2002). As such, 100 % complementarity between the siRNA seed region and an mRNA chicken gene was deemed unacceptable.

**Table 1:** Top sense-strand sequence of NP and PA siRNAs, the location of the target sequence in the corresponding NP or PA virus gene and siRNA specificity with the original AIV gene

Gene (bp region)	Sense strand sequence (5′–3′)
NP-siRNA	GGA TCT TAT TTC TTC GGA G-dTdT
H8N4 NP (1486–1504)	GGA TCT TAT TTC TTC GGA G
H6N2 NP (1453–1471)	GGA TCT TAT TTC TTC GGA G
PA-siRNA	GCA ATT GAG GAG TGC CTG A-dTdT
H8N4 PA (2077–2095)	GCA ATT GAG GAG TGC CTG A
H6N2 PA (2065–2083)	GCA ATT GAG GAG TGC CTG A

**Table 2:** The AIV strain and corresponding virus gene targeted by the NP or PA siRNA construct

Virus strain	Subtype	Gene	Accession
A/chicken/Texas/473-2/2010	H6N2	NP	*KM244053*
A/turkey/Colorado/235497/2003	H8N4	NP	*GU051910*
A/chicken/Texas/473-2/2010	H6N2	PA	*KM244051*
A/turkey/Colorado/235497/2003	H8N4	PA	*GU051912*

The shRNA expression plasmid, pmbv43, has been previously described (Buttaro and Fruehauf 2010). A total of 2 µg parent/pmbv43 plasmid (provided by Cambridge Biolabs, Cambridge, USA) was digested and subsequently treated with Alkaline Phosphatase, Calf intestinal (New England Biolabs, Ipswich, USA) and phenol/chloroform and ethanol precipitated. The resulting 8.4 kb linear parent/pmbv43 plasmid was gel extracted and isolated using dialysis.

The DNA template encoding the shRNA specific for NP and PA was: *Bam*HI site-sense sequence-hairpin loop (5′-TTC AAG AGA-3′)-antisense sequence-TTTTTTTTTT-*Sal*I site. Using standard cloning and plasmid purification methods, the NP/pmbv43 plasmid was commercially synthesized and purified (DNA2.0 Inc., Menlo Park, USA). Both strands of the PA DNA oligonucleotide sequence were commercially synthesized (Integrated DNA Technologies, Coralville, USA) and the PA/pmbv43 plasmid was generated in-house. The PA oligonucleotides were annealed, phosphorylated, and ligated into the linear pmbv43 plasmid. The resulting ligation mixture was transformed into DH5α competent cells and plated onto Luria Broth (LB) plates containing 10 µg/mL kanamycin (Kan). After incubating overnight, resulting colonies were screened by PCR. A single PCR positive PA/pmbv43 plasmid clone was purified using the PureLink HiPure Plasmid Maxiprep Kit (Life Technologies) and sequenced to verify proper PA/shRNA insertion.

NP/pmbv43, PA/pmbv43, and parent/pmbv43 were each transformed into CEQ221 competent *E. coli* cells (provided by Cambridge Biolabs) and plated onto Brain Heart Infusion (BHI) agar containing 25 µg/mL Kan and 50 µg/ml 2,3-Diaminopropionic Acid (BHI/Kan/Dap). Resulting colonies were screened by PCR, and for each, a single positive clone was sequence validated and propagated. Stocks were generated (OD_600_ = 1.0) and frozen back at −80 °C in 20 % glycerol. A single frozen aliquot from each vector stock was thawed for plate enumeration. Briefly, a 1 mL aliquot was centrifuged for 5 min at 5000 ×*g* and re-suspended in 1 mL of PBS containing 100 µg/mL Dap (PBS/Dap). The resulting vectors were serially diluted and plated in duplicate on BHI/Kan/Dap agar. Colony counts at each dilution were averaged to calculate overall CFU/mL and represented a viable concentration for vector stocks of anti-AIV/NP, anti-AIV/PA, and parent/pmbv43/CEQ (anti-AIV/scramble). This system allowed a live inoculum stock to be directly used in all future assays.

### Analysis of β(1) integrin expression in LMH cells

Avian tissues represent a novel target for tkRNAi, therefore, it was important to verify the presence of β(1) integrin receptors on the surface of LMH cells under normal conditions (uninfected) and post AIV infection. Total RNA was extracted from uninfected (normal growth conditions) and LMH cells infected six and 24 hpi with H8N4 virus. Chicken β-actin was used as an internal control for β(1) integrin expression. Primers and cycling conditions for chicken β-actin and β(1) integrin were previously published (Caprile et al. 2009). First strand cDNA was synthesized and conventional PCR amplification was completed using a 25 µL reaction containing: 0.075 µg of cDNA, 0.8 mM dNTPs (total), 1.6 mM MgCl_2_, 2.5 µL 10× Amplitaq Gold Buffer II, 0.8 U Taq DNA polymerase (Life Technologies), and 0.4 µM of each primer. PCR products were analyzed by 2 % agarose gel electrophoresis.

### Optimal tkRNAi vector concentration

LMH cells were seeded in 24-well plates 1 day prior to invasion to reach 80 % confluency. Anti-AIV/scramble vector was serially diluted 1:4 in invasion medium (Waymouth’s, 2 mM l-glutamine, 50 µg/ml of Dap) from 5 × 10^7^ to 7.8 × 10^5^ CFU/mL. Cells were washed twice with invasion medium before 1 mL of anti-AIV/scramble vector at each dilution was added to an appropriate well, in triplicate, and allowed to incubate for 2 h. Each plate included an untreated well (Waymouth’s complete medium) and a mock-invasion well (invasion medium only). After 2 h incubation, invasion medium was aspirated and wells were washed twice before fresh Waymouth’s complete medium was replaced. At multiple time points post invasion (24–48 h), vector cytotoxicity was assessed using visual signs of CPE and crystal violet staining as described above in “*cells and viruses*”, compared to the untreated control wells. The maximum anti-AIV/scramble vector concentration allowable, without inducing CPE, was selected for all subsequent invasion assays in LMH cells.

### Intracellular uptake of tkRNAi vectors

Using standard transformation methods, the anti-AIV/scramble vector was co-transformed with the red fluorescent protein (RFP) prokaryotic expression vector, pE2-Crimson (Clontech, Mountain View, USA), into CEQ221 competent *E. coli* cells as previously described (Bevis and Glick 2002) and live inoculum stocks were generated and enumerated. LMH cells were seeded in 8-well chamber slides 1 day prior to invasion. On the day of vector invasion, a 1 mL aliquot of the RFP-vector was prepared and serially diluted 1:4 in invasion medium starting at 5 × 10^7^ and ending at 7.8 × 10^5^ CFU/mL. LMH cells were prepared for invasion and 1 mL of RFP-vector at each dilution was added. After 2 h, wells were rinsed twice to remove unbound RFP-vector and fresh complete medium was replaced. To visualize RFP expression using fluorescent microscopy, cells were rinsed twice with PBS and fixed using ProLong Gold Antifade Reagent with 4′,6-diamidino-2-phenylindole (DAPI) (Life Technologies). Images were captured using the Eclipse Ti inverted fluorescent microscope (Nikon Instruments Inc., Melville, USA) at 40× magnification. DAPI stained nuclei associated with RFP signal were noted. To verify intracellular RFP-vector invasion and determine invasion efficiencies, LMH cells were detached at 20 and 36 h post invasion using trypsin and centrifuged at 500 ×*g* for 5 min. Supernatant was removed and cells were re-suspended in 2 mL cold PBS containing 5 % FBS. Intracellular RFP fluorescence was detected in the red (592–671/39 nm) channel by flow cytometry. Acquisition and analysis was performed on a Beckman Coulter MoFlo Astrios Flow Cytometer using Summit Software 6.1. One untreated (Waymouth’s complete medium), one mock-invasion control (invasion medium only) and one non-RFP invasion control well (anti-AIV/scramble vector at 3.1 × 10^6^ CFU/mL) was always included.

### Anti-AIV vector invasion and virus infection

LMH cells were seeded one day prior to invasion in 24-well plates to allow cell monolayers to reach 80 % confluency. On the day of vector invasion, 1 mL stock aliquots of anti-AIV/NP, anti-AIV/PA, and anti-AIV/scramble vector were thawed, centrifuged, and re-suspended in PBS/Dap. Vectors were diluted and the LMH cells were prepared for the invasion assay. Triplicate wells of the above vectors plus a cocktail of anti-AIV/NP + anti-AIV/PA vector (anti-AIV/cocktail) were prepared. Control wells were also included in triplicate (mock-invasion and untreated). After 2 h incubation, LMH cells were washed and fresh Waymouth’s complete medium was replaced.

Twenty four hours post vector invasion the LMH cells were prepared for infection by removing the growth medium and washing with inoculation medium (IM) containing 0.25 μg/mL L-1-Tosylamide-2-phenylethyl chloromethyl ketone (TPCK) treated trypsin (Sigma-Aldrich, St. Louis, USA), 3 % bovine serum albumin (BSA) (Gemini Bio-Products, Sacramento, USA), 2 mM l-glutamine, 100 Units/mL penicillin and 1 mg/mL streptomycin in Waymouth’s MB. The wash was removed and IM containing either H8N4 or H6N2 (MOI of 0.01) was added. Plates were incubated on a rocking platform for 1 h before removing the virus and replacing with fresh IM. Cell culture supernatants were harvested 48 hpi, centrifuged at 10,000 ×*g* for 10 min at room temperature, and frozen at −80 °C. For each virus tested, samples, including positive controls (AIV infected), negative controls (untreated LMH cells), and mock-invasion controls (mock-invasion + AIV infected) were included in triplicate wells.

### Evaluation of infectious virus titer

LMH cell culture supernatants containing infectious virus were quantitated by TCID_50_ on MDCK cells and titer was expressed as the TCID_50_/mL. Briefly, confluent monolayers of MDCK cultures growing in 96 well plates were inoculated with 100 µL/well of virus supernatant that was ten-fold serially diluted in MEM/EBSS, 2 mM l-glutamine, 3 % BSA, 0.5 % sodium pyruvate, 0.5 % MEM-NEAA, 100 U/mL penicillin and 1 mg/mL streptomycin, containing 1 µg/mL TPCK treated trypsin. Each dilution from 1:10 to 1:10^12^ was added in triplicate. At 48 hpi cells were fixed and stained with 0.1 % crystal violet and wells with CPE were scored as positive for virus growth. The TCID_50_/mL was calculated by the Reed and Muench mathematical technique (Reed and Muench 1938).

### Statistical analysis

All invasion experiments were repeated three times and on three different days. Due to observed changes in viral titers between different experimental days (data not shown), multiple linear regression analysis was used to adjust for variability between experimental days and compare adjusted mean log_10_ TCID_50_/mL values between vector treated and positive control (PC) samples (p < 0.05). Statistical analyses were performed using the data analysis and statistical software STATA 10 IC (StataCorp, College Station, USA).

## Results

### Design of effective shRNAs

BLAST screening revealed a lack of sequence complementarity (0 %) between the NP and PA siRNA seed regions and any known chicken gene. The wide range of genetic variation among different strains and subtypes of AIV makes it difficult to design siRNAs that can remain effective as antivirals. Therefore, it is important to design siRNAs targeting highly conserved sequences observed across many different strains, including HPAI viruses. Both siRNA sequences had 100 % alignment against >10,000 type A influenza viruses, including those isolated from swine, avian, equine, canine, and humans. These query matches included pathogenic H5, H9, and H7 subtypes isolated as recently as 2015. The potency of these two unique siRNA sequences has been extensively evaluated in previous work using traditional transfection techniques (Abrahamyan et al. 2009; Ge et al. 2003, 2004; Khantasup et al. 2014; Li et al. 2005; Sui et al. 2009; Tompkins et al. 2004).

### Verifying intracellular uptake and optimal vector concentration in avian cells

**Fig. 2:**
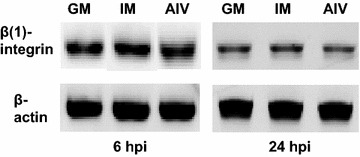
mRNA expression of β-actin and β(1) integrin in LMH cells cultured in normal growth medium (GM), inoculation medium without virus (IM), or infection medium six or 24 hpi with H8N4 virus

**Fig. 3:**
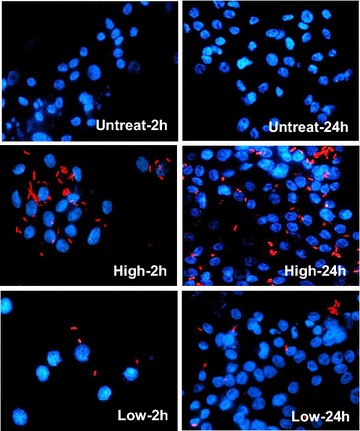
Vector uptake by chicken LMH cells assessed at two and 24 h post invasion with the anti-AIV/scramble vector tagged with RFP. LMH cells incubated with the RFP-vector at two doses (high = 7.8 × 10^5^ CFU/mL and low = 1.95 × 10^5^ CFU/mL). Nuclei were stained with DAPI. Magnification, X40

**Fig. 4:**
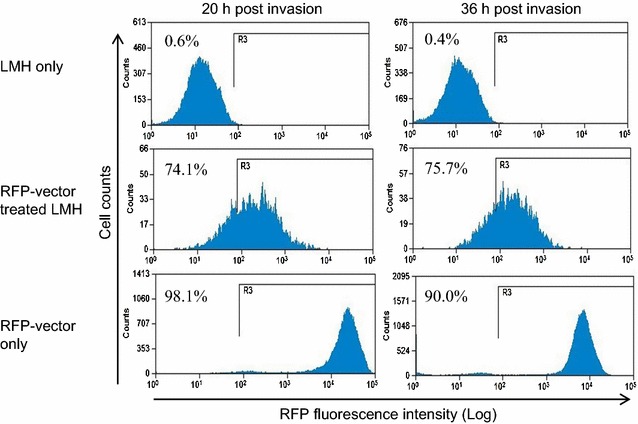
Flow cytometry data for LMH cells not treated with the RFP-vector (LMH only), LMH cells stably expressing RFP-vector (RFP-vector treated LMH), and RFP-vector only. LMH cells were treated with 7.8 × 10^5^ CFU/mL RFP-vector and intracellular RFP expression was assessed at 20 and 36 h post vector invasion. RFP fluorescent signal was normalized to the RFP-vector only expression and invasion efficiencies (%) are depicted as number of cells expressing RFP out of the total number of cells counted

The use of chicken primary hepatocellular carcinoma epithelial cells was not intended to represent a model for epithelial cells of the avian respiratory system. The tkRNAi system is not specific for respiratory mucosal epithelia; it broadly targets all mucosal epithelia. While tkRNAi delivery has been well documented in mammalian mucosal epithelial cells, this study reports the first time tkRNAi has been evaluated in avian cells. LMH cells were chosen to broadly represent an avian tissue model to verify that chicken β(1) integrin recognizes and interacts with invasion, the antigen expressed by the tkRNAi delivery vector and required for intracellular uptake. Moreover, LMH cells were utilized for this initial in vitro proof of concept work because they represent a commercially available chicken cell line with good reproducibility, they are specifically intended for transfection studies, they represent an appropriate avian tissue cell for AIV infection (Shinya et al. 1995; Swayne and Pantin-Jackwood 2006), and as epithelial hepatocytes, they represent appropriate mucosal epithelial cells to test tkRNAi vector uptake. Therefore, the expression of β(1) integrin in LMH cells when uninfected and post AIV infection was validated (Fig. 2). The maximum anti-AIV/scramble concentration allowable, without inducing CPE indicative of cytotoxicity, was 7.8 × 10^5^ CFU/mL. Therefore, all subsequent experiments adopted this vector concentration. Fluorescent microscopy demonstrated intracellular expression of the RFP-vector in LMH cells at two and 24 h post invasion (Fig. 3). Flow cytometry further verified intracellular RFP-vector expression at 20 and 36 h post invasion with 7.8 × 10^5^ CFU/mL. The tkRNAi invasion efficiencies in LMH cells were calculated to be 74.1 and 75.7 %, respectively (Fig. 4). Together, these results indicated the tkRNAi delivery platform was appropriate for avian cells.

### Anti-AIV vector antiviral activity in avian cells

Previous work has demonstrated inhibition of viral NP and PA mRNA transcripts using RT-qPCR following transfection with the same 19nt NP and PA siRNA constructs utilized in this work (Abrahamyan et al. 2009; Ge et al. 2003; Khantasup et al. 2014; Li et al. 2005; Sui et al. 2009). The objective of this current work was to show inhibition of infectious viral particle shedding. RT-qPCR does not differentiate between infectious and non-infectious virus, however, the TCID_50_ assay allows for the quantification of infectious viral particles. Therefore, given how extensively the potency of these siRNAs have been validated using RT-qPCR in avian and mammalian models against AIV and to aid in demonstrating the efficacy for inhibiting infectious virus, LMH cell culture supernatants containing shed virus were quantitated by TCID_50_ on MDCK cells and infectious titer was expressed as the TCID_50_/mL. There were no statistically significant differences in adjusted mean viral titers between PC and mock-invasion samples (data not shown); therefore, PC samples were used to make statistical comparisons to the vector treated samples. The 95 % confidence interval (CI) for the adjusted mean viral titers were calculated and represent the magnitude of variability between viral titers on different experimental days and across replicates (Table 3). The adjusted mean viral titers between anti-AIV vector treated and untreated PC samples were compared across all experimental days (Table 3). These results indicated the level of antiviral activity was more pronounced against the H8N4 virus. However, in all experimental sets and for both H8N4 and H6N2, the cocktail vector resulted in significantly lower viral titers compared to the corresponding PC titers, with log_10_ reductions ranging from 1.9–4.0.

**Table 3:** Anti-AIV vector protection in chicken LMH cells as measured by log_10_ and fold-reductions in viral shedding titers compared to untreated controls

Sample	*n*	Adjusted mean^a^ (treated)	95 % CI (treated)	Adjusted mean (PC)	95 % CI (PC)	*P* value^b^	Log_10_ reduction^c^	Fold reduction^d^
H8N4 virus MOI 0.01								
Anti-AIV/NP	9	1.1	(0.0, 2.6)	4.9	(3.7, 6.2)	<0.001	3.8	6310
Anti-AIV/PA	9	3.0	(2.1, 3.8)	5.5	(4.6, 6.4)	<0.001	2.5	316
Anti-AIV/cocktail	9	0.9	(0.0, 2.1)	4.9	(3.9, 6.0)	<0.001	4.0	10,000
Anti-AIV/scramble	10	2.8	(2.0, 3.5)	4.9	(4.1, 5.8)	<0.001	2.1	126
H6N2 virus MOI 0.01								
Anti-AIV/NP	9	4.7	(3.1, 6.2)	6.3	(4.8, 8.0)	0.043	1.6	39.8
Anti-AIV/PA	9	4.9	(3.6, 6.2)	6.3	(4.8, 7.9)	0.031	1.4	25.1
Anti-AIV/cocktail	9	4.4	(3.0, 5.9)	6.3	(4.9, 7.8)	0.013	1.9	79.4
Anti-AIV/scramble	9	5.5	(4.6, 6.3)	6.3	(5.3, 7.4)	0.053	0.8	6.3

Mean viral titers were adjusted by day, using experimental day as a fixed effect

^a^Expressed as mean log_10_ TCID_50_/mL adjusted by day

^b^Comparing adjusted mean log_10_ TCID_50_/mL from treated to untreated PC samples (p < 0.05)

^c^Log_10_ reduction in mean infectious titer compared to untreated control

^d^Fold reduction in infectious titer (geometric mean) compared to untreated control

## Discussion

RNAi approaches have been used to demonstrate the antiviral activity of siRNAs against influenza viruses and report various degrees of viral inhibition in multiple subtypes tested (Ge et al. 2003, 2004; Jiao et al. 2013; Khantasup et al. 2014; Li et al. 2005; Stoppani et al. 2015; Tompkins et al. 2004; Zhou et al. 2007, 2008). In one study, siRNAs targeting NP and PA inhibited the lab-adapted PR8 virus (Ge et al. 2003). In mice experiments, NP and PA siRNAs/shRNAs have inhibited multiple type A influenza subtypes, including H5N1 and mouse-adapted H1N1, H7N7 and H9N2 (Ge et al. 2004; Tompkins et al. 2004; Zhou et al. 2008). A recent siRNA study, targeting NP, reports inhibition against AIV (H7N1) and swine influenza (H1N2, H3N2, and H1N1 strains) (Stoppani et al. 2015). Critically, in all of these studies, viral reductions represent those associated with siRNA/shRNA silencing using traditional RNAi delivery approaches. Our work represents the first time tkRNAi has been assessed both as an shRNA delivery vehicle to avian cells and as an antiviral for influenza virus. If the in vitro success of this antiviral validated in this current work can be demonstrated with future in vivo studies, this antiviral technique has the potential to attain clinical relevance by providing a safe and efficacious method of delivering siRNAs to target tissues, including avian epithelium, without inducing cytotoxicity. Nonrelated work has successfully used this RNAi delivery approach to target cancer genes (Buttaro and Fruehauf 2010; Kruhn et al. 2009; Xiang et al. 2006; Zhao et al. 2005). Cequent Pharmaceuticals (now Marina Biotech, Bothell, WA, USA) recently developed a therapeutic based on the tkRNAi platform (CEQ508), which is currently in clinical testing for the treatment of Familial Adenomatous Polyposis in humans.

As was demonstrated in our work and in previous studies, there were variations in the level of viral inhibition observed when the same siRNA construct was used against different AIVs. This can be explained in several ways. It is possible that in different AIVs these target mRNA sequences correspond with slightly different functions and might interact differently with host factors or interact with entirely separate host factors, all resulting in slightly altered siRNA potency. Directly measuring anti-AIV vector knockdown of NP and PA could help explain the observed variation in antiviral activity between viruses. However, our outcome of interest was the ability of these anti-AIV vectors to inhibit infectious viral shedding. As such, quantitating infectious viral titers using the TCID_50_ assay was an appropriate end-point analysis for this work. Additionally, including the anti-AIV/scramble vector served as a method of differentiating NP and PA shRNA antiviral activity associated with targeting the NP and PA viral genes from any non-specific viral inhibition caused by the bacterial vector itself. In all experiments, and for both viruses tested, antiviral activity associated with the NP and PA specific anti-AIV vectors was more potent compared to the scramble vector alone.

The scramble vector had less profound antiviral activity, but it did reduce infectious AIV titers. There is a possible explanation for this observation. The innate immune response is part of the host’s early defense mechanism. Toll-like receptors (TLRs) play a key role in the innate response by recognizing and binding bacterial components, including endotoxins like lipopolysaccharide (LPS), which act as immune enhancers (Bessler et al. 1990). Nonpathogenic *E. coli* bacteria deliver these anti-AIV vectors; therefore, it is not surprising that the extracellular presence of bacterial endotoxins would be detected, likely via TLR-4 recognition (Schoen et al. 2004). In this case, LPS could act like a vaccine adjuvant, thereby stimulating the innate response prior to AIV infection and mounting an additional level of viral protection.

This work was necessary and provided in vitro proof-of-concept demonstrating both successful delivery and the antiviral potential of these novel anti-AIV vectors against AIV in an avian cell model. Further work is needed to determine if these antiviral vectors would reduce AIV shedding in poultry. As such, findings from this work have been translated into ongoing efforts aimed at assessing efficacy in experimentally challenged chickens. This novel antiviral could represent a more effective and universal control method for AIV in poultry. These vectors were engineered for targeted siRNA delivery to mucosal epithelium, including respiratory tissues, and could be applied to large populations of birds using a spray or nebulization technique. Therefore, this antiviral approach could represent a scalable and clinically applicable technology for avian and human influenza and a prototype for RNAi-based antivirals against other viruses that infect mucosal tissues.
